# Strategies to Strengthen Iraq's Primary Healthcare System: A Systematic Literature Review With Special Focus on Society 5.0

**DOI:** 10.1002/puh2.70033

**Published:** 2025-02-06

**Authors:** Fakher Rahim, Karlygash Toguzbaeva, Arsen Aidaraliev, Kenesh Dzhusupov

**Affiliations:** ^1^ International Medical Faculty Osh State University Osh Osh Region Kyrgyzstan; ^2^ Department of Public Health Asfendiyarov Kazakh National Medical University Almaty Kazakhstan; ^3^ International University of Kyrgyzstan Bishkek Kyrgyzstan; ^4^ Department of Public Health International Higher School of Medicine Bishkek Kyrgyzstan; ^5^ Department of Public Health Osh State University Osh Kyrgyzstan

**Keywords:** health system, primary healthcare (PHC), Society 5.0

## Abstract

**Background and Aims:**

The primary healthcare (PHC) system is a comprehensive, equitable, and efficient approach to supporting health and social well‐being by offering prevention, treatment, and rehabilitation services close to living environments. The PHC system in Iraq is currently facing issues that limit its effectiveness and efficiency. This research aims to discover strategies to enhance the PHC system in Iraq.

**Methods:**

We conducted a systematic search for articles on initiatives aimed at enhancing the PHC system, covering the period from 1980 to June 2024, using eight databases and Google Scholar. Included articles that met the criteria were analyzed with RevMan 5.3 software.

**Results:**

A total of 18,705 articles were extracted. After removing duplicates and items without full text, 15,971 studies remained for title and abstract review, of which 14,175 were removed. Finally, 18 research articles related to PHC strengthening were used. Data collection methods included checking databases (12 studies), interviews (6 studies), focus groups (3 studies), questionnaires, and archival data (15 studies).

**Conclusion:**

In the context of Society 5.0, smart technology's practical applications in healthcare aim to enhance decision‐making, patient care, and service delivery. Smart technologies can help policymakers and health administrators make clinical decisions in complex care situations and streamline procedures like paperwork. This ultimately will improve the quality and effectiveness of healthcare services by providing accurate, timely, and personalized information to support decision‐making. When rebuilding and strengthening the PHC system, addressing the historical, social, cultural, and economic variables is important.

**Trial Registration:**

Not applicable.

## Graphical abstract

Graphical Abstract

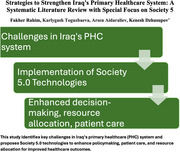

## 1 Introduction

The health system includes individuals, groups, and organizations responsible for policymaking, financing, generating resources, and delivering services to promote health [1]. Primary healthcare (PHC), recognized by the World Health Organization (WHO) in 1975, aims to provide accessible, comprehensive, and efficient health services [2]. In 1978, the WHO and the United Nations Children's Fund organized an international meeting in Alma‐Ata, Kazakhstan, which outlined a plan for PHC to achieve the goal of “health for all.”[3].

PHC is defined as essential health services that are affordable and available to the general population throughout their lifespan, including health education, illness prevention, treatment, rehabilitation, and palliative care [4]. The key elements of PHC encompass health education, improved nutrition, access to safe drinking water and environmental enhancement, maternal and child health promotion, immunization, availability of essential medications, management of prevalent diseases and injuries, and communicable disease prevention [5]. Its fundamental principles include access, community engagement, health promotion, the appropriate skills and technology, and cross‐sector collaboration. PHC is characterized by accessibility, comprehensiveness, coordination, and continuity [6].

The Alma‐Ata Declaration identified health as a fundamental human right and emphasized global cooperation to achieve universal PHC. Nations implementing PHC have observed significant benefits, improved health outcomes, reduced mortality rates, and increased patient satisfaction. A 2024 World Bank report estimated that PHC initiatives could address 90% of health issues, with only 10% requiring specialized hospital services. Countries with robust PHC systems have better service accessibility [7], improved treatment quality [8], decreased disease incidence [9], fewer hospitalizations [10], lower mortality rates [11], increased patient satisfaction [7], reduced healthcare costs [12], and improved population health equity [13, 14]. For example, efficient PHC initiatives in Europe have led to better health outcomes, improved equity, and decreased hospitalization rates [15]. A study in 29 European countries found superior health outcomes for individuals with chronic conditions in nations with a robust and well‐integrated PHC system [16]. In 2018, WHO reaffirmed its commitment to expanding primary care to achieve sustainable development and universal health coverage [17].

Despite these successes, PHC faces challenges, including variability in professional training, diverse care scopes, limited infrastructure, and inconsistent resource allocation. Certain programs can address these gaps and strengthen the PHC system [18].

Artificial intelligence (AI) is a transformative technology simulating human intelligence in machines, particularly in healthcare [19, 20]. Iraq's PHC system has achieved improvements in service availability, decreased maternal and child mortality, and increased life expectancy, [21, 22]. However, it faces significant challenges, including structural rigidity, inadequate and uneven resource allocation, limited technology use, insufficient staffing and motivation, weak information systems, centralized decision‐making, inconsistency between departments, and inadequate supervision. Administrative bureaucracy and a lack of transparency and accountability are associated with evaluation [23, 24]. This has diminished its efficacy and efficiency in disease management, particularly in epidemics and pandemics like COVID‐19 [25, 26].

In this context, the emergence of Society 5.0 offers a transformative vision for addressing these challenges. Introduced in Japan in 2016, the concept of Society 5.0 in the Fifth Fundamental Plan of Science and Technology, aims to integrate advanced technologies, including AI, the Internet of Things (IoT), and robotics, into societal frameworks to balance economic progress with solutions to social issues [27, 28, 29]. Society 5.0 provides a pathway for integrating smart technologies to enhance decision‐making, patient care, and service delivery [30]. By leveraging these technologies, health systems can become more adaptive, efficient, and patient‐centered, addressing challenges unique to low‐ and middle‐income countries like Iraq.

AI, a pivotal component of Society 5.0, has revolutionized medical diagnostics, administrative efficiency, telemedicine, and personalized medicine by enabling precise medical image interpretation, predictive analytics, and streamlined data processing [21]. However, challenges persist, including data privacy, system integration, ethical concerns like algorithmic transparency, and the human element in care [31, 32]. Future advancements will focus on ethical integration to enhance chronic disease management and mental health treatment [33, 34].

Therefore, this study *aims* to identify strategies to strengthen Iraq's PHC system within the framework of Society 5.0. By evaluating the current state, examining current barriers, proposing actionable reforms, and exploring the role of smart technologies, the research seeks to provide policymakers and healthcare administrators with evidence‐based insights. Ultimately, this work aspires to contribute to a resilient and responsive PHC system in Iraq that aligns with the evolving demands of its population and leverages the transformative potential of Society 5.0 technologies.

## 2 Methods

The study used a systematic literature review methodology to analyze and clarify data, revealing the relationships between the health intervention and outcomes. This review utilized a comprehensive seven‐step systematic literature review protocol [35]. The protocol involved the following steps:

1. *Formulating research questions* that guide the review. For this study, the primary research focus was on identifying strategies to enhance Iraq's PHC system.
2. *Theoretical framework*. Our theoretical framework involves understanding Iraq's current PHC system, challenges, and potential interventions to improve its effectiveness and efficiency.
3. *Search strategies*. Relevant English keywords were used (e.g., Primary Health Care [Title/Abstract]) OR (Primary Care [Title/Abstract]) OR (Primary Healthcare [Title/Abstract]) AND (Iraq [Text Word]), across databases like Web of Science, Scopus, PubMed, and Embase, covering publications from 1980 to 2024.

Inclusion and exclusion criteria: Studies addressing strengthening PHC until the end of June 29, 2024, were included. Non‐English articles, studies published after the cutoff date, or sources lacking full text were excluded.

1. *Evidence collection*. Comprehensive database searches, added by gray literature via Google Scholar, were organized in the EndNote program to manage the citations.
2. *Evidence evaluation*. Articles were screened in two steps: two authors (F.R. and K.D.) independently screened titles and abstracts, followed by full‐text reviews conducted by the other two authors (T.K. and A.A.), resolving discrepancies.
3. *Data analysis*. Using Ritchie and Spencer's framework, data were categorized into themes like governance, financing, and human resources [36]. To aid in the data, we employed RevMan 5.3 software to facilitate synthesis and thematic analysis. We reported the findings using the PRISMA‐ScR checklist [37].
4. *Refining the proposal submission program's theory*. We identified strategies and interventions to strengthen Iraq's PHC system, focusing on necessary and sufficient resources, innovative technologies, and effective organizational structures.

### 2.1 Society 5.0

Society 5.0 emphasizes the necessity of developing society as well as the societal implications of technology [38]. To achieve Society 5.0's goal, it is necessary to reconsider both the relationships between society and technology, as well as the relationships between individuals and society facilitated by technology (Table 1).

**TABLE 1:** Design, scope, objective, and key elements of Society 5.0.

Society 5.0			
Design	Scope	Objective	Key elements
The 5th Science and Technology Basic Plan and the Comprehensive Strategy on Science Technology, and Innovation	Super‐smart society	Society as a whole	The integration of the internet and physical space at a sophisticated level Striking a balance between economic progress and addressing social problems A society that prioritizes the needs and well‐being of individuals

## 3 Results

**FIGURE 1:**
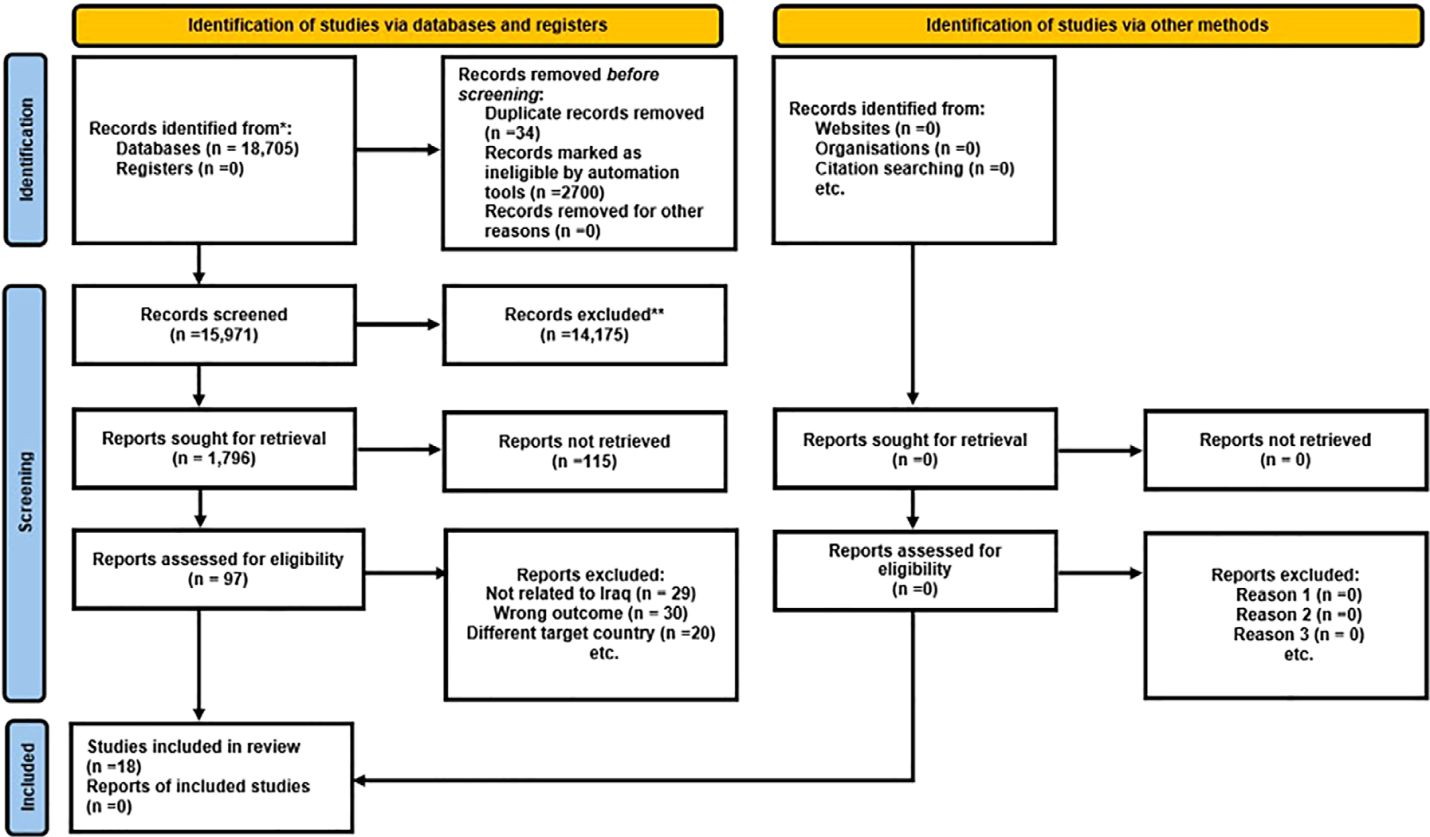
PRISMA 2020 flow diagram for new systematic reviews that included searches of databases, registers, and other sources.

In total, 18,705 articles were extracted through databases. After removing duplicates and items without full text, 15,971 studies remained. Screening of titles and abstracts excluded 14,175 articles, leaving 1796 articles for full‐text review. The number of 115 articles in the field of strengthening PHC was obtained at this stage. After careful study of the remaining articles, 97 articles were removed due to lack of application in the field of strengthening PHC. No articles were found by checking the sources of the articles. Finally, 18 research articles related to the strengthening of PHC were used in this study (Figure 1).

Out of them, there were:

observational studies—8 studies (44.4%),
quantitative method—6 studies (33.3%),
combined method—1 study (5.5%), and
qualitative method—3 studies (16.67%) (Table 2).

**TABLE 2:** Characteristics of included studies.

Study ID	Method	Study population	Location	Findings
Al‐Uzri et al. [39]	WHO mhGAP‐IG 2.0 training for primary care physicians	17 participants from different medical specialties	Baghdad, Iraq	WHO mhGAP‐IG 2.0 training improved primary care physicians’ knowledge in Baghdad
Washi et al. [40]	Evaluation of the quality assurance for school health services	171 respondents (6 directors of PHC centers, 32 health and nursing staff, and 133 school principals)	Al‐Numaniya District, Iraq	Quality assurance for school health services in Al‐Numaniya was moderate in all essential components, such as structure, process, and outcome
Mahmood and Saleh [41]	A cross‐sectional study using a questionnaire‐based survey	A cluster of 6 households includes 2400 individuals	Erbil, Iraq	Patient satisfaction in Erbil requires integration and improvement of service quality components
Abdulrahman et al. [42]	A cross‐sectional design	403 parents and 62 HCPs from 20 PHCCs	Duhok, Iraq	Collaboration among healthcare facility departments and staff needs improvement in Duhok
Nguyen et al. [43]	A convergent mixed‐methods study design, quantitative and qualitative questionnaires	123 clients, 26 providers, 40 non‐MH staff, and 12 directors provided data	Sulaymaniyah, Iraq	A mPixed methods study showed supportive integration experiences in Sulaymaniyah
Amily et al. [44]	An interventional study	42 vaccinators from 10 PHCs	Wasit, Iraq	Training PHC's vaccinators improved immunization practices in Wasit
Khan et al. [45]	A framework based on five components	535,253 individuals spread over 55 camps 513,978 individuals from 247 camps	Baghdad, Iraq	Framework assessment indicated high‐quality care in Baghdad camps
Shabila et al. [46]	Q‐methodology for eliciting subjective viewpoints and identifying shared patterns among individuals	40 persons PHC providers	Erbil, Iraq	Sharing PHC service complaints can enhance the system in Erbil
Shabila et al. [47]	Q‐methodology for eliciting subjective viewpoints and identifying shared patterns among individuals	40 primary healthcare providers representing 8 PHC centers	Erbil, Iraq	Knowledge of the opinions held by PHC providers regarding the system may improve its functionality
Burnham et al. [48]	Household national survey	A cluster of 10 households of 7 public sector PHC facilities in each of 17 of Iraq's 18 governorates	Baghdad, Iraq	Lower income individuals predominantly utilize public sector PHC Centers as crucial healthcare providers in Baghdad
Shabila et al. [24]	A qualitative study using a self‐administered questionnaire survey	46 primary care managers, public health professionals, and academics	Erbil, Iraq	Found significant barriers to PHC services, including system organization and management concerns, a shortage of and poor medications, and unbalanced staffing and experience distribution
Sadik et al. [49]	Multistage evaluation using a pre‐ and post‐test questionnaire	PHC providers from 143 centers	Baghdad, Babil, Basrah, Dewaniyah, Karbala, and Najaf	There is a notable transformation, not just in understanding, but also in the subsequent display of practical abilities by trained professionals
Burnham et al. [50]	Household national survey	A cluster of 10 households of seven public sector PHC center	Baghdad, Iraq	Lower‐income individuals predominantly utilize public sector PHC Centers as crucial healthcare providers
Noël et al. [51]	Pilot study conducted a cross‐sectional survey	37 PHC providers	Baghdad, Iraq	PHC providers should be aware of common post‐deployment health conditions
Boscarino et al. [52]	Need assessment of PHC providers’ knowledge and awareness	1200 patients and 3600 of their family members	Baghdad, Iraq	Significant mental health gaps discovered
Al‐Zwaini et al. [53]	A questionnaire survey	50 persons PHC providers	Ramadi, Iraq	Needs for improving the knowledge and training
Godichet and Ghanem [54]	A questionnaire survey	10 people in all 4 PHC center	Mosul, Iraq	obvious need to explore ways to adapt profiles of medical consumption in the PHC centers
Hashim et al. [55]	A structured questionnaire interviews	500 patients and 500 healthcare workers selected from 250 PHC centers	Baghdad, Iraq	The national tuberculosis (TB) program has significantly enhanced the understanding

By data collection methods, there were:

database reviews—12 studies,
Interviews—3 studies,
Focus groups—3 studies (Table 2).

Geographically, the majority of the studies (44.45%) were conducted in Baghdad, the capital city of Iraq.

This study revised the basic theory of the PHC program by identifying its strategies, field factors, and effective mechanisms. The final theory of the PHC program states: “The PHC system has lofty goals and appropriate strategies, equipped with necessary and sufficient resources (financial, human, physical, and information) and appropriate and innovative technologies, organized in the form of competent teams.” Skilled and committed multispecialty with specific roles and tasks for health workers, who provide comprehensive, quality, and affordable healthcare coordinated with other health services (intervention program), in case of strengthening the elements of governance and leadership, financing, human resources, equipment and medicine, information systems and provision of health services and optimal adaptation to the surrounding political, economic, social and technological environment (field), increase the satisfaction, commitment, accountability and responsibility of managers and health workers, trust participation and satisfaction of patients and people (a mechanism) and finally, securing, maintaining and improving the health of the people of the society (result).

The data were analyzed using Ritchie and Spencer's framework analysis approach, resulting in the identification of several key themes:

‐ Governance and leadership: Challenges in organization, communication, and inter‐sectoral cooperation.
‐ Financing: Need for improved allocation and utilization of resources.
‐ Human resources: Insufficient staffing and motivation among health workers.
‐ Technology: Limited adoption of modern technologies and information systems.
‐ Medicine and equipment: Shortage and uneven distribution.
‐ Health services delivery: Need for better service delivery and patient satisfaction.

Thematic analysis resulted in the following implications for practice:

‐ AI and smart technologies: Potential to improve decision‐making, patient care, and operational efficiency.
‐ Community engagement: Essential for the sustainability and effectiveness of PHC interventions.
‐ Training and development: Continuous education and training programs are necessary for health workers.

## 4 Discussion

This study aimed to identify strategies to strengthen Iraq's PHC system in the context of Society 5.0. Strengthening the PHC system plays a significant role in increasing universal health coverage, increasing access to health services and benefiting from them, improving the quality of health services, reducing the costs of the health system and paying out of people's pockets, improving justice in health, reducing the rate of illness and death, and promoting community health. The findings highlight significant insights into governance, financing, human resources, technology, medicine, and service delivery. By comparing the challenges faced by Iraq's PHC system with findings and recommendations from the selected articles, the study contextualizes these issues and potential solutions.

In this study, 18 published works describing strengthening the PHC system were identified and categorized into six areas: current status of Iraq's PHC, governance and leadership, financing, human resources, technology, medicine and equipment, information systems, and providing health services.

### 4.1 Current Status of PHC in Iraq

The PHC system in Iraq faces numerous challenges that limit its effectiveness and efficiency [21, 22, 23, 27, 28, 29]. These include insufficient governance and leadership, uneven resource allocation, inadequate staffing, limited adoption of modern technologies, and shortages of medical supplies. The existing health information systems are fragmented and underutilized, impeding evidence‐based decision‐making. Additionally, centralized decision‐making and weak inter‐sectoral collaboration hinder adaptive and coordinated responses to public health needs. The reliance on traditional healthcare delivery methods and the absence of innovative technologies further exacerbate the system's inefficiencies.

Despite these challenges, Iraq possesses certain enablers that could support the adoption of Society 5.0 activities. These include a growing interest in digital transformation within healthcare, the availability of skilled professionals in urban areas, and international partnerships aimed at improving health outcomes. However, these enablers remain underdeveloped and require strategic investment to unlock their potential.

### 4.2 Governance and Leadership

Effective governance requires structured management mechanisms, including communication, inter‐sectoral cooperation, policy formulation and planning, supervision and leadership, and monitoring and evaluation [56]. Governance challenges in Iraq's PHC system include issues in organization, communication, inter‐sectoral cooperation, and policy formulation [24, 40, 42, 46, 49]. The reviewed articles emphasize that governance processes benefit significantly from transparency, accountability, and stakeholder engagement. For example, collaborative governance models in African countries have demonstrated improved service delivery through community engagement and inter‐sectoral collaboration. [57]. Similarly, introducing mechanisms by the Ministry of Health such as performance monitoring and public reporting can address inefficiencies in Iraq [58]. This includes establishing an appropriate structure for services such as family physicians and home healthcare, as well as improving the referral system and promoting collaboration among different departments. Actively encouraging their engagement is one way to increase the involvement of private and non‐profit organizations in planning and management. Furthermore, promoting individual responsibility and self‐care, as well as evaluating the health system and publicly announcing performance indicators, can help to improve overall healthcare.

To adapt to Society 5.0, Iraq's governance systems need to incorporate AI‐driven decision‐support systems to enhance transparency and streamline policy. Training programs for health leaders to effectively utilize these tools are also essential [24]. Lessons from Nigeria emphasize the importance of involving local communities in decision‐making process to enhance trust and ensure contextually relevant interventions [59].

### 4.3 Resource Allocation and Financing

The review underscores the critical importance of equitable resource allocation and innovative financing mechanisms in Iraq's PHC system [24, 46, 48]. Active society involvement in the PHC system enhances its long‐term viability and efficient utilization of healthcare services. Understanding the social, economic, and cultural determinants that impact a society's health will result in the most suitable healthcare program being implemented. Evidence from Africa demonstrates that community engagement significantly improves service accessibility and delivery [60]. A Nigerian study suggests that allowing communities, as primary beneficiaries, to take the lead in health interventions enhances relevance and sustainability [61]. Iraq's PHC system, which suffers from uneven resource distribution, could benefit from these approaches. AI and big data analytics, as proposed in Society 5.0, offer tools to predict resource needs and optimize allocations, ensuring underserved areas receive adequate support. [29]. Performance‐based financing, observed in other settings, could also incentivize efficiency and service quality. To implement these changes, Iraq must establish robust digital infrastructures and promote cross‐sectoral collaboration to ensure the successful deployment of AI‐based tools.

### 4.4 Human Resource Management

Insufficient staffing and low motivation among health workers remain major challenges in Iraq [39, 42, 43, 44, 45, 46, 49, 55]. Continuous education and training in evidence‐based practices are essential for all PHC staff, including managers, doctors, and nurses. In Africa, the training of non‐physician health workers has improved access to healthcare in rural areas [62].

AI‐powered training platforms can enhance skillsets and motivation among healthcare workers. Robotic assistants and AI tools can augment the workforce, reducing the burden on human resources and improving service efficiency [62]. Furthermore, insights from Nigeria highlight the value of empowering healthcare workers to design and implement contextually appropriate interventions, which can boost morale and effectiveness [61].

### 4.5 Technology and Information Systems

Limited adoption of modern technologies and weak information systems hinder Iraq's PHS system [24, 39, 40, 42, 49, 54, 55]. Reliable and prompt information is critical for decision‐making by policymakers and managers [63]. Developing integrated information management systems with robust data collection, analysis, exchange, and utilization capabilities can address these gaps. For example, implementing the electronic health record (EHR) system improved diabetes management in China [64].

Society 5.0 promotes the integration of IoT devices, AI, and advanced information systems to enhance real‐time patient monitoring and data management [28, 64].

However, adopting these emerging healthcare technologies requires addressing new legal, ethical, and organizational challenges, including data privacy and energy constraints [65].

### 4.6 Medicine and Equipment

There is a shortage and uneven distribution of medical supplies and equipment in the PHC system in Iraq [39, 40, 54]. AI‐driven supply chain management systems can predict demand, optimize inventory, and ensure the timely distribution of medical supplies and equipment, thereby addressing shortages and distribution issues [66]. Wearable IoT devices play a vital role in delivering uninterrupted, instantaneous patient monitoring and can enhance patient monitoring and precision medicine, improving diagnostic accuracy and care delivery.

### 4.7 Health Services Delivery

Improving service delivery and patient satisfaction is vital [24, 40, 42, 46, 54, 55]. Technologies like telemedicine, AI‐assisted diagnostics, and personalized medicine can improve service delivery by providing remote consultations, accurate diagnoses, and tailored treatment plans, enhancing overall patient satisfaction [19, 30]. Society 5.0's proactive and accessible healthcare approach can transform patient care, enabling precision medicine and advanced modelling through AI and machine learning.

### 4.8 Practical Applications and Ethical Considerations

The general public will have access to research‐derived medical information. AI algorithms will tailor this information, often without any regulatory oversight from the healthcare system. AI algorithms can support clinical decisions in complex care situations, reducing errors and improving patient outcomes [33]. AI and robotics can streamline procedures like documentation, allowing healthcare professionals to focus more on patient care [31]. This illustrates the concept of the “democratization of healthcare.” The primary obstacle to the widespread adoption of IoT is the presence of data that has the potential to infringe on human rights and core values, such as privacy, security, and safety. This problem is particularly prevalent in countries that have limited resources [66]. The integration of AI and smart technologies raises ethical issues, including data privacy, algorithmic transparency, and the potential loss of the human element in healthcare. Addressing these concerns is crucial for the ethical adoption of Society 5.0 principles [32]. Additionally, mistrust in healthcare systems, as observed in Nigeria, underscores the importance of transparency and trust‐building in introducing new technologies [67].

## 5 Limitations

The study primarily focuses on Iraq's PHC system, which may have unique challenges and conditions that are not applicable to other countries. The findings and proposed strategies might not be directly transferable to other regions with different healthcare infrastructures, economic conditions, or cultural contexts. This limits the ability to generalize the results to a broader global context. The study relies on available literature, which may include biases, incomplete data, or varying levels of methodological rigor. The quality and comprehensiveness of the data can influence the reliability of the findings. Incomplete or biased data sources may lead to skewed conclusions, potentially overlooking important factors or misrepresenting the effectiveness of certain strategies.

The integration of Society 5.0 technologies, such as AI and IoT, is discussed in theoretical terms without extensive empirical evidence from within Iraq. Therefore, the practical implementation of these technologies in Iraq's PHC system may face unforeseen challenges, such as infrastructure limitations, resistance to change, or ethical concerns. This uncertainty can impact the feasibility and effectiveness of the proposed strategies.

The study uses a systematic literature review, which, although comprehensive, is limited by the scope of the selected articles and the criteria for inclusion. The exclusion of non‐English articles and the specific time frame may result in the omission of relevant studies, potentially affecting the breadth and depth of the review. This could lead to incomplete conclusions or a lack of consideration for recent developments and innovative approaches outside the reviewed literature.

The ethical implications of AI, including data privacy, algorithmic transparency, and the potential loss of the human element in healthcare, are acknowledged but not deeply explored. Overlooking these considerations can lead to ethical dilemmas and practical challenges in implementing AI‐driven solutions. Failure to address these issues comprehensively may hinder the acceptance and success of integrating smart technologies into the PHC system.

It is critical to establish clear and specific rules and laws for the advancement and implementation of AI in the healthcare industry. This will guarantee the ethical generation of AI technology that aligns with societal needs. Systems use data and algorithms to make recommendations; nevertheless, these recommendations may not fully understand a patient's specific needs and preferences. AI's ability to deliver physical care is limited. AI systems cannot administer medication or perform medical procedures, which are essential components of nursing and midwifery care. AI systems may have a limited capacity to handle complex circumstances, such as those experienced by healthcare staff, which require critical thinking and decision‐making abilities. Intelligent systems use precise and detailed data to make recommendations. Data quality issues, such as insufficient or erroneous data, might limit the accuracy of AI suggestions. The cost of developing and implementing smart technologies may limit their availability and adoption in particular healthcare areas. Guidelines and regulations are required to ensure safety and transparency when using AI in healthcare. Intelligent technologies in healthcare necessitate the collection of personal and sensitive data, which requires protection against cyberattacks and the preservation of patient confidentiality. Accountability is critical to the development and deployment of smart technologies in the healthcare business.

### 5.1 Implication for Policymakers, Practitioners, and Researchers

Policymakers need to develop robust governance structures that foster inter‐sectoral collaboration, transparent decision‐making, and accountability. Effective leadership training programs should be established to enhance the capabilities of healthcare managers and leaders.

Efficient allocation and utilization of financial resources are crucial. Policymakers should focus on equitable distribution of resources to ensure all regions have access to quality healthcare.

To predict healthcare needs and optimize resource distribution, they should utilize big data analytics. The introduction of performance‐based financing will incentivize improvements in healthcare delivery.

Investing in continuous education and training for healthcare workers is essential to maintain a skilled workforce. Policymakers should address motivational issues and ensure adequate staffing levels. Implementing AI in developing training programs will provide personalized learning experiences. The introduction of incentives and career development opportunities will boost motivation and retention among healthcare workers.

The integration of modern technologies, such as AI, IoT, and EHRs, can significantly enhance healthcare delivery. Policymakers must ensure the necessary infrastructure and regulatory frameworks are in place. Pilot AI and IoT projects in selected PHC centers must be implemented to evaluate their impact and scalability. To ensure data privacy and security while using these technologies, regulatory guidelines should be established.

Enhancing the delivery of health services is critical for patient satisfaction and overall system effectiveness. Practitioners should adopt patient‐centered approaches and leverage technology for better service delivery. To improve access to care, especially in remote areas, telemedicine services can be used. AI‐assisted diagnostics and personalized medicine will enhance the accuracy and efficiency of treatments.

Future research can be focused on empirical validation of the effectiveness and feasibility of integrating AI and smart technologies in Iraq's PHC system. To assess the long‐term impact of proposed strategies on health outcomes and patient satisfaction, longitudinal studies can be carried out. The development of comprehensive ethical and regulatory frameworks for the use of AI in healthcare, ensuring data privacy, transparency, and the preservation of the human element, is another important research topic.

To find strategies for fostering greater community involvement in the planning and implementation of PHC services, the role of community engagement in enhancing the effectiveness and sustainability of PHC interventions should be explored.

At last, it is important to investigate the impact of smart technologies on health disparities and access to care, particularly in marginalized populations. Future research must answer such questions as how AI and telemedicine services affect health outcomes in economically disadvantaged communities, what are the barriers to technology adoption in low‐income areas, and how can they be overcome.

## 6 Conclusion

The health system in Iraq is currently facing significant challenges, including the escalating burden of chronic non‐communicable diseases, the emergence of novel diseases like COVID‐19, and the rise of antimicrobial resistance. Hence, health system policymakers must shift their focus from disease treatment to prevention. Strengthening the PHC system is crucial for improving population health and reducing illness and death rates. A one‐size‐fits‐all approach cannot be applied to enhance the PHC system in various countries. When rebuilding and strengthening the PHC system, it is essential to address the historical, social, cultural, and economic variables that impact it. A systematic strategy, grounded in systems thinking, is essential.

Effective PHC requires robust governance, sustainable financing, efficient information management, and high‐quality service delivery. Synchronizing PHC reforms with broader health system reforms is critical to addressing ageing populations and chronic disease burden, enhancing collaboration and coordination within the health system.

Integrating smart technologies from Society 5.0 into healthcare can improve decision‐making, patient care, and system efficiency. AI can support clinical decisions, streamline administrative tasks, and enhance diagnostic accuracy, though it cannot replace the human elements of care. Ethical considerations, such as data privacy and compassionate care, must guide the use of AI. Smart technologies offer innovative ways to provide healthcare, helping practitioners learn more effectively and deliver better services. By leveraging these advancements, Iraq can build a resilient and responsive PHC system that aligns with Society 5.0 and meets the healthcare needs of its population.

## Author Contributions

**Fakher Rahim**: conceptualization, methodology, software, data curation, formal analysis, project administration, writing–original draft; supervision. **Karlygash Toguzbaeva**: formal analysis, data curation, resources, writing–review and editing, software, validation. **Arsen Aidaraliev**: writing–review and editing, visualization, data curation, software, validation. **Kenesh Dzhusupov**: conceptualization, methodology, validation, investigation, data curation, formal analysis, writing–review and editing, project administration.

## Disclosure

The opinions, findings, and conclusions or recommendations expressed in this material are those of the author(s) and do not necessarily reflect the views of affiliated institutions.

## Ethics Statement

This is a systematic review with no study participants.

## Conflicts of Interest

Kenesh Dzhusupov is an Editorial Board member of the *Public Health Challenge Journal* and a co‐author of this article. To minimize bias, he was excluded from all editorial decision‐making related to the acceptance of this article for publication.

## Permission to Reproduce Material From Other Sources

No material from other sources has been reproduced in this manuscript. All content is original and created by the authors.

## Data Availability

This study did not generate any new data. All relevant data are contained within the manuscript and its Supporting Information files.
